# Comparison of Resampling Methods and Radiomic Machine Learning Classifiers for Predicting Bone Quality Using Dual-Energy X-Ray Absorptiometry

**DOI:** 10.3390/diagnostics15020175

**Published:** 2025-01-14

**Authors:** Mailen Gonzalez, José Manuel Fuertes García, María Belén Zanchetta, Rubén Abdala, José María Massa

**Affiliations:** 1 Instituto de Investigación en Tecnología Informática Avanzada, Universidad Nacional del Centro de la Provincia de Buenos Aires, Tandil 7000, Argentina; jmassa@intia.exa.unicen.edu.ar; 2 Consejo Nacional de Investigaciones Científicas y Técnicas, Buenos Aires 1414, Argentina; 3 Departamento de Informática, Escuela Politécnica Superior, Universidad de Jaén, 23071 Jaén, Spain; jmf@ujaen.es; 4 Instituto de Diagnóstico e Investigaciones Metabólicas, Buenos Aires 1012, Argentina

**Keywords:** dual energy X-ray absorptiometry, trabecular bone score, radiomics, data resampling, machine learning

## Abstract

**Background/Objectives**: This study presents a novel approach, based on a combination of radiomic feature extraction, data resampling techniques, and machine learning algorithms, for the detection of degraded bone structures in Dual X-ray Absorptiometry (DXA) images. This comprehensive approach, which addresses the critical aspects of the problem, distinguishes this work from previous studies, improving the performance achieved by the most similar studies. The primary aim is to provide clinicians with an accessible tool for quality bone assessment, which is currently limited. **Methods**: A dataset of 1531 spine DXA images was automatically segmented and labelled based on Trabecular Bone Score (TBS) values. Radiomic features were extracted using Pyradiomics, and various resampling techniques were employed to address class imbalance. Three machine learning classifiers (Logistic Regression, Support Vector Machine (SVM), and XGBoost) were trained and evaluated using standard performance metrics. **Results**: The SVM classifier outperformed the other classifiers. The highest F-score of 97.5% was achieved using the Grey Level Dependence Matrix and Grey Level Run Length Matrix feature combination with SMOTEENN resampling, which proved to be the most effective resampling technique, while the undersampling method yielded the lowest performance. **Conclusions**: This research demonstrates the potential of radiomic texture features, resampling techniques, and machine learning methods for classifying DXA images into healthy or degraded bone structures, which potentially leads to improved clinical diagnosis and treatment.

## 1. Introduction

Osteoporosis is a bone disease characterised by a low bone mineral density (BMD) and structural deterioration, which leads to an increased fracture risk that mainly affects postmenopausal women [1]. Dual X-ray Absorptiometry (DXA) uses information from the radiation absorption at two different energies in a specific bone area to calculate the BMD or its standardised equivalent index, the T-Score, which is used to diagnose Osteoporosis [2].

Although BMD is a useful index for diagnosing and predicting fracture risk, it does not provide information about bone structure. It only represents 50–60% of fracture prediction, suggesting that factors other than bone mass determine bone strength [3].

Even though DXA images are 2D projections of 3D structures, which include radiography images (RX), some texture analysis methods allow for an indirect analysis of the bone trabecular microarchitecture [4] as the Trabecular Bone Score (TBS). This texture index evaluates DXA images using a grey-level difference measurement technique based on the application of variograms [5]. Several studies showed that there is a significant correlation between 3D studies such as X-ray microtomography (m-CT) and TBS [6,7]. In addition, it can be used for predicting osteoporotic fractures independently of bone densitometry results [8].

Range values of TBS have been established, with a TBS ≥1.350 being considered healthy; a TBS between 1.200 and 1.350 is considered partially degraded; and a TBS ≤1.200 defines a degraded microarchitecture [5].

The TBS is a first step towards quantifying the quality of bone microstructure from spine DXA images, and nowadays, it is used by the medical community as an additional indicator when assessing fracture risk [9]. Nevertheless, its utilisation is constrained by the fact that it is distributed as proprietary software, which is costly, particularly for developing countries. In addition, it is only available for specific models of DXA equipment.

Radiomics is a technique for extracting detailed quantitative and textural features from medical images, which help to reveal tissue characteristics and variations that might escape human observation. Beyond this, the use of standardised and well-established features, which are validated over time across various imaging modalities, facilitates subsequent model interpretability and its behaviour in machine learning applications. This approach has shown significant success in detecting and describing diseases, especially those related to vascular, bone, and tumour detection [10,11]. Due to the large amount of data generated by radiomics, Machine Learning (ML) techniques have become useful for handling these data, allowing them to address classification in most medical imaging problems.

Although osteoporosis is a significant disease in terms of population incidence, it is generally expected that the healthy group of patients will be the majority [12]. Consequently, the balance of the data becomes relevant, leading us to contemplate the use of data resampling techniques. Furthermore, these methods have recently been considered not only for the balance per se but also for data enhancement in medical applications [13,14].

To the best of our knowledge, no research has been published that employs radiomic indicators validated with TBS metrics. However, some related studies can be highlighted due to the use of radiomics and ML to classify samples based on their BMD values or to predict fracture risk [15,16,17,18,19]. In particular, ref. [15] analyses images of the same modality with similar radiomic features and ML classification methods. However, using BMD as a label, their study reports a lower performance compared to the results achieved in this work.

The main objective of this study is to classify DXA images into groups representing healthy or degraded bone structure, based on the TBS value ranges. For this, radiomic texture indicators are used as features alongside ML techniques. Additionally, well-established resampling methods are employed to address the problem of imbalanced datasets. The proposed ML approach in this work has the potential to be adaptable to future technological advances in medical imaging equipment, which may improve the DXA image properties such as resolution, contrast, and depth colour, among others. On the other hand, non-learning-based methods, like the current TBS, cannot completely adapt to these changes.

## 2. Materials and Methods

### 2.1. Data Acquisition

The retrospective study population comprises 1531 patients aged 46 to 76 years. The spine raw DXA images were obtained using a Lunar Prodigy Advance (GE Healthcare, Madison, WI, USA) and the TBS was calculated using TBS iNsight version 3.0.2.0. The spatial resolution of the selected images is about 300×280 pixels.

The study set comprises one raw image per patient, of which 13% have a TBS value belonging to a degraded microstructure, and the rest have a range associated with a healthy microstructure. The balance between the sample groups is consistent with the incidence of the disease in the population [12,20]. DXA images and TBS values were obtained from postmenopausal women who periodically consulted to Instituto de Diagnóstico e Investigaciones Metabólicas (IDIM), a bone health research centre in Buenos Aires. Images were collected between 2018 and 2022, considering as an inclusion criterion whether it was possible to evaluate the set of vertebrae of the lumbar spine.

The dataset was partitioned into a training set comprising 70% of the samples and a testing set containing the remaining 30%. The training set included 1071 samples, of which only 130 belonged to the target class degraded. Conversely, the testing set comprised 460 samples, with 404 labelled as healthy and 56 as degraded.

### 2.2. Image Segmentation and Feature Extraction

**Figure 1:**
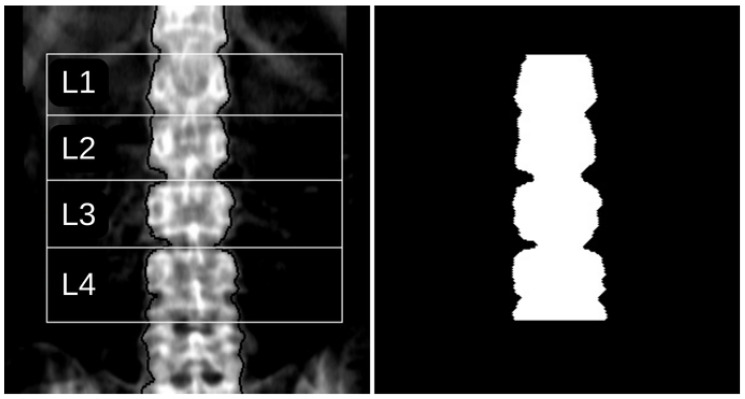
The left panel displays a DXA image with ROIs defined by the equipment’s software, and the right panel shows the corresponding automatically generated mask. In the left figure, the lateral contour is delineated by black pixels, while white lines individually divide the four vertebrae of interest.

The region of interest (ROI) in this study, corresponding to the lumbar vertebrae L1 to L4, was defined based on the automatic segmentation provided by the DXA equipment’s enCORE v17 software platform. This segmentation was validated by a specialised technician. To ensure consistent results, it is essential to use the same ROI for subsequent analysis. A binary mask was generated using contour detection techniques to replicate the segmentation provided by the software corresponding to the ground truth. Figure 1 illustrates an example of a DXA image with the ROIs defined by the enCORE v17 software and its corresponding binary mask.

The mask generation algorithm was specifically designed to accurately detect the boundaries of the lumbar spine, focusing on the vertebrae L1 to L4, to create a binary mask that preserves the pixels identified by the enCORE v17 software. The algorithm consists of the following steps: (1) Identifying the left contour of the spine; (2) Locating the pixels corresponding to the right contour of the spine, both defined by black pixels; (3) Identifying the four vertices of the rectangle enclosing the L1–L4 lumbar vertebrae; (4) Generating the binary mask, where the pixels within ROI are white. An unstructured description of each step is provided below.

To locate the left contour (step 1), the algorithm scans the image along the Y-axis from top to bottom. For each Y-coordinate, it evaluates pixels along the X-axis from the left edge to the middle of the image: if a pixel has a value of 0, checks are performed to determine whether it belongs to the image background or the ROI. This involves assessing the pixel’s neighbourhood, ensuring that the mean intensity value of the right pixels is greater than the mean value of the left pixels. Additionally, the algorithm verifies the connectivity of detected pixels along the Y-axis, as the contour forms a connected segment in any of the eight possible directions. A similar process is applied for locating the right contour (step 2), following the same logic for detection.

To identify the vertices (step 3), the algorithm scans from the four edges of the image towards the centre to locate the horizontal and vertical white pixel segments that define the L1–L4 rectangle. Once these segments are identified, the corresponding Y coordinates are recorded to determine the upper boundary of the L1 vertebra and the lower boundary of the L4 vertebra.

Finally, in step 4, a totally black image with the same dimensions as the original image is created, and pixels identified in steps 1, 2, and 3 are assigned a white value. The region enclosed by these pixels is also filled with white, defining the ROI in the binary mask, as required by the Pyradiomics library. An example of the resulting binary mask is shown in Figure 1.

Raw images and masks were employed as input to an algorithm for extracting radiomic features, which utilises the open-source Python package PyRadiomics 3.0 [21]. A comprehensive set of 93 statistical features was extracted, which are organised into different categories according to the radiomic method from which they were derived. Specifically, 18 features were obtained from First Order Statistics (FOIS), 24 from the Grey Level Co-occurrence Matrix (GLCM), 16 from the Grey Level Run Length Matrix (GLRLM), 16 from the Grey Level Size Zone Matrix (GLSZM), 14 from the Grey Level Dependence Matrix (GLDM), and 5 from the Neighbouring Grey Tone Difference Matrix (NGTDM). As a result, six datasets were created, each corresponding to the features derived from a specific method: FOIS-DS, GLCM-DS, GLRLM-DS, GLSZM-DS, GLDM-DS and NGTDM-DS.

The Pyradiomics shape features, which are calculated based on the mask geometry, were excluded from the analysis as they are more closely associated with overall bone morphology and are significantly influenced by cortical bone rather than the trabecular microarchitecture of interest.

While dimensionality reduction techniques can be applied to combine features to find a representative subset, this may affect the subsequent interpretability of each feature [22]. For this reason, it was decided to combine them manually, and among the many possible combinations, three concatenations of selected feature sets were chosen. One dataset was formed with the whole set of 93 features (WFS), and the other two datasets were generated based on the good performance of some of the features in previous works, such as [23]. One is formed by the concatenation of GLDM-DS and GLRLM-DS named as GLDM-GLRLM-DS, and the other consists of the concatenation of GLDM-DS, GLRLM-DS and GLSZM-DS), named as GLDM-GLRLM-GLSZM-DS.

In summary, nine datasets were created and used in this work: the six Pyradiomics feature collections and the three combinations.

### 2.3. Data Resampling

The initial dataset exhibited a pronounced imbalance in the number of samples between the two target classes, a common challenge in clinical datasets that can significantly impair the performance and generalisation capacity of machine learning models. Initial experiments using this imbalanced dataset demonstrated suboptimal results, underscoring the necessity of mitigating this issue. Several resampling techniques were applied specifically to the training set to mitigate this imbalance and improve predictive accuracy, which initially comprised 1071 samples (942 healthy and 129 degraded). By resampling the class distribution within the training set, the aim was to ensure a more equitable representation of both classes during model training.

To increase the representation of the minority class, oversampling methods were employed, specifically the Synthetic Minority Over-sampling Technique (SMOTE) [24] and Adaptive Synthetic Sampling (ADASYN) [25]. SMOTE generates synthetic instances by interpolating between existing samples of the minority class, while ADASYN focuses on generating more diverse synthetic examples in regions where the decision boundaries are less distinct. On the other hand, random undersampling (UNDERSAMPLING) was employed to reduce the number of samples in the majority class by pseudo-randomly removing instances, thereby achieving a more balanced dataset.

Furthermore, two hybrid techniques that combine oversampling and undersampling were examined for their effectiveness in dataset balancing. SMOTETomek [26] combines SMOTE with Tomek-Links, a method that identifies and removes ambiguous samples from the majority class to sharpen the decision boundary. The second approach, SMOTEENN [27], integrates SMOTE with the Edited Nearest Neighbour (ENN) technique, which removes noisy and potentially misclassified samples from the majority class to enhance class separation. Both of these techniques have been widely validated in clinical contexts, demonstrating their utility in improving the quality and predictive power of imbalanced datasets [13].

Resampling methods were applied to the training dataset using the open-source Python package Imbalanced-learn, version 0.11.0. For this study, the hyperparameter values for the resampling methods were kept at their default settings, as is commonly defined in many studies [28,29]. This decision was based on two primary considerations.

Firstly, the default configurations in Imbalanced-learn have been established through empirical testing, often providing robust and generalisable results across a variety of domains. By adhering to these defaults, we aimed to minimise any potential biases introduced by manual tuning and to establish a baseline that reflects the standard behaviour of the resampling techniques.

Secondly, the focus of this study was not on fine-tuning individual resampling methods but rather on comparing their relative effectiveness in balancing datasets and improving model performance. Therefore, using default parameters ensured that the comparison remained fair and consistent, allowing the evaluation to focus on the inherent capabilities of each resampling technique rather than the influence of parameter settings. This approach provides a starting point for future studies, where hyperparameter optimisation can be explored.

Following the application of these resampling methods, new datasets were generated in which the feature values of the synthetic augmented samples were constrained within the range of the original sample values. This ensured that the synthetic instances closely mirrored the distribution of the initial data, thereby maintaining the dataset’s inherent characteristics.

**Figure 2:**
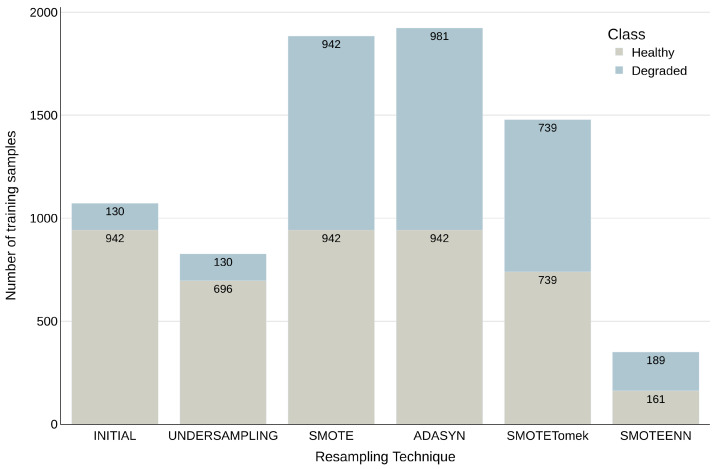
Resampled Class Distribution in the WFS Dataset. Each bar represents a resampling technique, showing the final number of samples for each class. The INITIAL bar indicates the original, imbalanced distribution.

Figure 2 presents the resulting class distribution for the WFS dataset to illustrate the impact of resampling techniques on class balance. Each bar corresponds to a specific resampling method and displays the count of samples for both classes. The INITIAL bar represents the original, imbalanced dataset. The visual comparison of the bars highlights the performance of each technique in achieving a more balanced class distribution.

**Figure 3:**
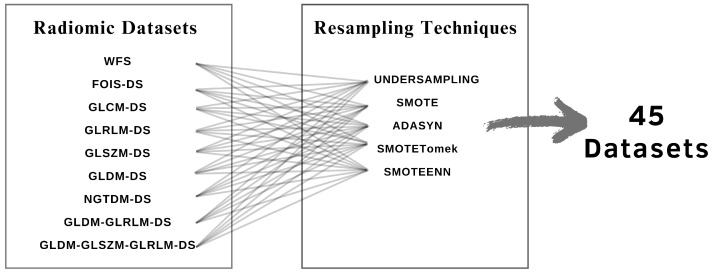
Application of five resampling techniques to each radiomic dataset results in 45 new, more balanced datasets.

Resampling methods were applied to the training sets of each radiomic dataset, resulting in the creation of 45 distinct datasets, as illustrated in Figure 3. As a result of these resampling techniques, most of the training sets were adequately balanced, with the exception of those that underwent undersampling.

### 2.4. Machine Learning Classification

Three widely used machine learning classifiers were employed to analyse the 45 generated datasets: Logistic Regression (LR), Support Vector Machine (SVM), and XGBoost or Extreme Gradient Boosting (XGB). These classifiers were selected based on their effectiveness in similar research contexts, such as predicting fracture risk and classifying osteoporosis using DXA and RX images [30,31,32].

As previously mentioned, the dataset was divided into training and test sets. Resampling techniques were applied exclusively to the training set to improve class balance, and these adjusted datasets were subsequently used for model fitting and training. The reserved test sets contain only original, non-synthetically generated samples, allowing for a more reliable and realistic evaluation of the employed methodology.

A 5-fold cross-validation technique was employed to optimise hyperparameters and train the machine learning models. A grid search approach was used to systematically explore potential hyperparameter values for each algorithm. All possible combinations of the defined hyperparameters were explored to find the optimal configuration that maximised the mean F-score measure for each classifier. The F-score was selected as the evaluation metric due to the imbalance in some datasets, which could make accuracy an inadequate measure of classification performance.

The SVM and LR models and training strategies were implemented using the scikit-learn library in Python. For the SVM classifier, three kernel types were explored: linear, polynomial, and radial basis function (RBF). The regularisation parameter, denoted as C, was varied over a wide range, from $10^{-5}$ to $10^{5}$, allowing for flexibility in controlling the decision boundary. The gamma parameter, which affects the influence of individual data points, was tested with values ranging from $2^{-2}$ to $2^{5}$, as well as the options auto and scale.

In the case of XGB, the learning rate was tested with values 0.01, 0.1, 0.3, and 1.0, as this parameter controls the step size at each iteration, balancing the trade-off between training speed and convergence. For the maximum depth of the trees, values 4, 6, 8, and 10 were evaluated, which influence the model’s ability to capture complex patterns while managing the risk of overfitting. Additionally, the number of estimators, representing the number of boosting rounds, was explored with values 1, 10, 50, 100, and 500, allowing the model to balance underfitting and overfitting by varying the total number of weak learners combined into the final ensemble.

For the LR classifier, three types of penalties were considered: L1, L2, and ElasticNet. The regularisation parameter C was again varied from $10^{-5}$ to $10^{5}$, impacting the strength of the regularisation applied. Additionally, various solvers were evaluated, including liblinear, saga, and lbfgs.

All other hyperparameters, unless explicitly specified, were maintained at their default values as defined by their respective libraries.

While a variety of hyperparameter combinations were examined, only those that yielded optimal training outcomes for each dataset and resampling method were presented due to space limitations. Detailed settings are provided in Appendix A, which includes three tables: Table A1, Table A2 and Table A3. Each table lists the datasets that achieved the best training performance with RL, SVM and XGB classifiers, respectively. For each classifier, the selected hyperparameters, along with the mean and standard deviation of the F-score across the five folds, are reported. This presentation ensures a concise yet comprehensive overview of the optimal configurations based on their performance metrics.

### 2.5. Resulting Pipeline

**Figure 4:**
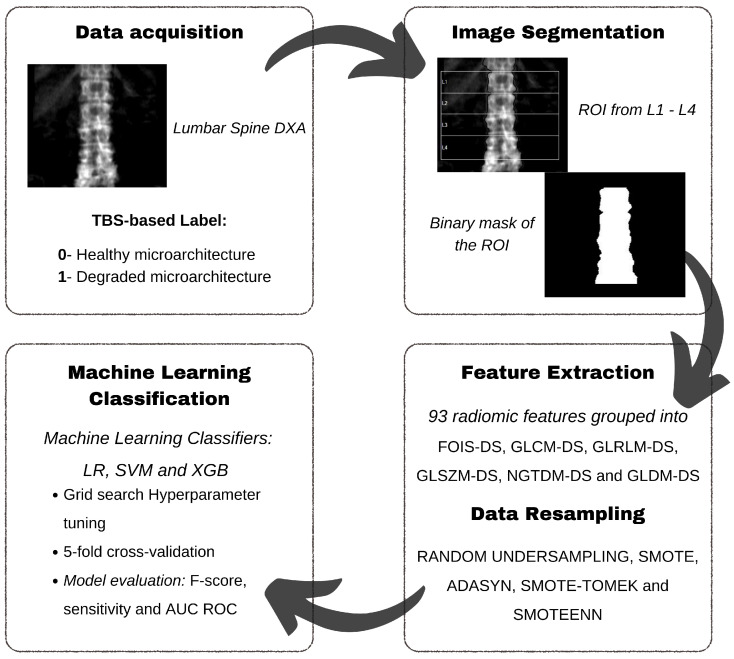
Work pipeline: (1) Image acquisition and labelling as healthy or degraded; (2) Image segmentation and binary mask generation; (3) Feature extraction using Pyradiomics; (4) Resampling techniques application; (5) ML classification using the proposed classifiers.

An overview of the proposed methodology is illustrated in the pipeline shown in Figure 4. Following data acquisition, raw images are automatically segmented to generate ROI masks, which are used for radiomic feature extraction. The training partitions of extracted features were synthetically resampled to create more balanced datasets. Finally, classification is performed using machine learning algorithms. The project’s codebase was organised following this pipeline, and the repository can be requested from the corresponding author.

## 3. Results

This section presents the evaluation results of SVM, LR and XGB classifiers across the 45 datasets generated. After training, models were evaluated, and metrics such as accuracy, sensitivity, precision, F-score and AUC ROC were calculated for each case.

Table 1 summarises the F-score, AUC ROC and sensitivity for each individual feature collection and resampling technique, highlighting the best-performing classifier in terms of F-score. At first sight, SVM outperformed the other classifiers in most datasets, followed by XGB and LR. Regarding datasets, the highest F-score results were 0.917 for GLCM-DS and SMOTEENN, 0.967 for GLRLM-DS and SMOTEENN, 0.912 for GLSZM-DS and SMOTE, 0.953 for GLDM-DS and SMOTEENN, 0.925 for NGTDM-DS and SMOTEENN, and 0.914 for FOIS-DS and ADASYN.

**Figure 5:**
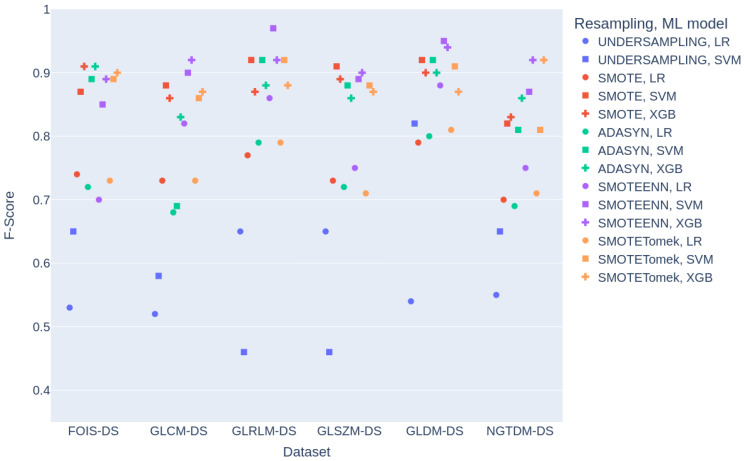
Classification results. The X-axis represents the dataset used for model training and testing, while the colour indicates the resampling technique. The symbol shape represents the ML model employed. The Y-axis displays the F-score value obtained in each case.

Furthermore, Figure 5 shows the classification results for all classifiers and individual feature collections, comparing the obtained F-score metric. To improve visualisation, the points for each dataset are separated to avoid overlap, as some combinations share identical F-score values. In the case of classification results obtained with XGB using datasets processed with the UNDERSAMPLING technique, all F-score values were below 0.25 and were therefore excluded from the figure. The figure highlights that certain feature sets, such as GLRLM-DS and GLDM-DS, demonstrate the best overall performance.

Datasets that employed UNDERSAMPLING as a resampling technique obtained lower performance, with F-score values ranging from 0.100 to 0.818. In contrast, datasets using SMOTEENN generally achieved the best performance with F-score values ranging from 0.758 to 0.967. Moreover, UNDERSAMPLING led to greater variability in F-score across classifiers.

**Figure 6:**
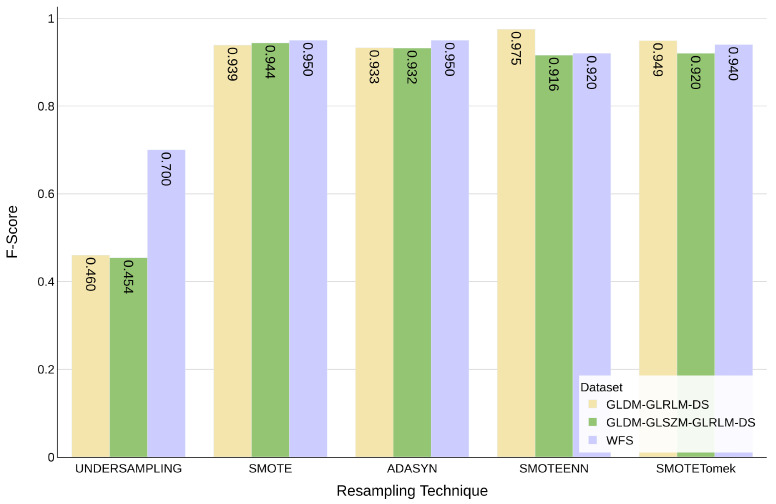
F-score comparison of SVM classifier performance on the three combined feature sets (WFS, GLDM-GLRLM-DS and GLDM-GLSZM-GLRLM-DS) using five different resampling techniques: UNDERSAMPLING, SMOTE, ADASYN, SMOTEENN and SMOTETomek.

Furthermore, as shown in Figure 6, the combined datasets with specific resampling techniques yielded high F-score: 0.950 for WFS and ADASYN, 0.975 for GLDM-GLRLM-DSand SMOTEENN, and 0.944 for GLDM-GLSZM-GLRLM-DS and SMOTE. In these cases, SVM was the best-performing classifier.

## 4. Discussion

This section presents a discussion of the results obtained regarding the feature sets and resampling techniques. Firstly, an analysis of the best-performing feature sets is presented, followed by a discussion on the impact of resampling techniques on classification performance.

The superior performance of the GLRLM-DS and GLDM-DS features compared to the other Pyradiomics features is reasonable considering that these features are similar to those that have been used successfully in previous related studies [5,33]. These features capture grey-level repetition patterns, which have shown strong correlations with bone quality indicators.

When the two top-performing feature sets (GLDM-DS and GLRLM-DS) were combined, the results surpassed those of other combinations, including individual feature sets. Notably, this combination also outperformed the WFS set, suggesting that certain features within the WFS superset might have downgraded the overall classification accuracy.

Regarding the resampling techniques, a greater dispersion between the F-score values is observed when UNDERSAMPLING is used. This may be due to the reduction in the number of samples, which increases intra-class variability, maintains class imbalance, and reduces classification performance.

By contrast, when the other resampling techniques were applied, the density of samples in the feature space increased due to the synthesis of new instances for the degraded class. The resampling techniques SMOTE, SMOTETomek, and SMOTEENN share the same oversampling strategy for generating samples of the minor class. In the case of SMOTETomek, there are not enough Tomek links between healthy and degraded classes to be deleted by this method. This is consistent with the fact that SMOTETomek performed almost as well as SMOTE.

The ENN strategy embedded in SMOTEENN, which removes samples of the healthy class close to samples of the degraded class, is more aggressive than that of SMOTETomek. Consequently, SMOTEENN produces a dataset with more separated samples between classes and is expected to achieve the best result.

In clinical practice, having a high true positive rate represented by the sensitivity metric is relevant, considering that degraded bone microarchitecture increases the fracture risk. The results show that, in most cases, there is a correspondence between high F-scores and high sensitivity values.

Although oversampling techniques work in the feature space of real samples in terms of limitations, they could generate samples with feature vectors, which cannot be produced by hypothetical real samples. Considering that the training and validation labels have an associated error, it can be propagated to the method proposed in this work. In addition, the dataset could contain hidden biases regarding the patient’s medical conditions, such as metabolic diseases or treatments that affect the bone texture.

As previously mentioned [15], the most comparable study, reported a maximum AUC ROC of 0.780, which is notably lower than the highest AUC ROC of 0.994 achieved in this work.

## 5. Conclusions

This study presented a comprehensive strategy for the classification of DXA samples into healthy and degraded classes, based on their TBS range values. To achieve this, a radiomic approach utilizing Pyradiomics was employed to extract diverse feature sets from the DXA images, some of which were combined to improve classification performance. The results highlight the importance of GLRM-DS and GLDM-DS feature sets, which were identified as particularly representative of bone texture and yielded superior classification results.

To address the inherent class imbalance in the dataset, we evaluated several resampling techniques: UNDERSAMPLING, SMOTE, ADASYN, SMOTETomek, and SMOTEENN. Among these, SMOTEENN was identified as the best approach based on the balance and classification performance. Finally, three machine learning classifiers (SVM, LR, and XGB) were employed for classification. Grid search and cross-validation techniques were utilised to identify the optimal combination of hyperparameters. Subsequently, the models were trained and evaluated using each of the generated datasets. Overall, SVM consistently outperformed the other classifiers.

Among the evaluated combinations, the SVM classifier, which was trained on the dataset GLRLM-GLDM-DS and balanced using the SMOTEENN technique, achieved the highest F-score of 97.5%. This result demonstrates the potential of radiomic texture features, resampling techniques, and machine learning for classifying DXA images into healthy and degraded bone structures, potentially leading to improved clinical diagnosis and treatment.

To further optimise the proposed approach, future work will focus on a more in-depth analysis of the GLRLM-DS and GLDM-DS feature sets. An ablation study will be conducted to identify the most impactful features within these sets, enabling feature selection and potentially improving the model’s efficiency and interpretability. Additionally, we plan to investigate the use of automated features extracted from convolutional neural networks to compare the similarity and performance of handcrafted radiomic features and deep learning-based approaches.

To enhance model generalisation and clinical applicability, efforts will be made to expand the dataset by including a larger number of real-world DXA samples, thus reducing reliance on synthetic data. Furthermore, incorporating samples from a partially degraded class will refine the model’s ability to distinguish subtle variations in bone health, enabling more precise clinical assessments.

## Figures and Tables

**Table 1:** Summary of results obtained from testing ML classifiers on each individual radiomic dataset.

Feature Set	Resampling Technique	Best Classifier	Sensitivity	F-Score	AUC ROC
FOIS-DS	UNDERSAMPLING	SVM	0.684	0.650	0.789
	SMOTE	XGB	0.916	0.906	0.963
	ADASYN	XGB	0.918	0.914	0.967
	SMOTETomek	XGB	0.901	0.899	0.960
	SMOTEENN	XGB	0.929	0.887	0.960
GLCM-DS	UNDERSAMPLING	SVM	0.579	0.579	0.688
	SMOTE	SVM	0.921	0.876	0.912
	ADASYN	SVM	0.804	0.847	0.921
	SMOTETomek	XGB	0.914	0.869	0.929
	SMOTEENN	XGB	0.970	0.917	0.961
GLRLM-DS	UNDERSAMPLING	LR	0.684	0.650	0.743
	SMOTE	LR	0.901	0.922	0.970
	ADASYN	SVM	0.903	0.919	0.977
	SMOTETomek	SVM	0.888	0.917	0.977
	SMOTEENN	SVM	0.987	0.967	0.994
GLSZM-DS	UNDERSAMPLING	LR	0.684	0.650	0.743
	SMOTE	SVM	0.907	0.912	0.965
	ADASYN	SVM	0.905	0.882	0.945
	SMOTETomek	SVM	0.915	0.885	0.948
	SMOTEENN	SVM	0.947	0.886	0.919
GLDM-DS	UNDERSAMPLING	SVM	0.947	0.818	0.875
	SMOTE	SVM	0.965	0.920	0.972
	ADASYN	SVM	0.911	0.918	0.973
	SMOTETomek	SVM	0.974	0.912	0.965
	SMOTEENN	SVM	0.977	0.953	0.986
NGTDM-DS	UNDERSAMPLING	SVM	0.684	0.650	0.713
	SMOTE	XGB	0.876	0.833	0.898
	ADASYN	XGB	0.869	0.833	0.918
	SMOTETomek	XGB	0.881	0.859	0.917
	SMOTEENN	XGB	0.943	0.925	0.974

## Data Availability

The datasets presented in this article are not readily available because they are in the process of being published with the medical institution involved.

## Abbreviations

The following abbreviations are used in this manuscript:

DXA	Dual Energy X-ray Absorpiometry
TBS	Trabecular Bone Score
BMD	Bone Mineral Density
m-CT	Microtomography
ML	Machine Learning
ROI	Region of Interest
FOIS	First Order Statistics
FOIS-DS	First Order Statistics dataset
GLCM	Grey Level Co-occurrence Matrix
GLCM-DS	Grey Level Co-occurrence Matrix dataset
GLRLM	Grey Level Run Length Matrix
GLRLM-DS	Grey Level Run Length Matrix dataset
GLSZM	Grey Level Size Zone Matrix
GLSZM-DS	Grey Level Size Zone Matrix dataset
GLDM	Grey Level Dependence Matrix
GLDM-DS	Grey Level Dependence Matrix dataset
NGTDM	Neighbouring Grey Tone Difference Matrix
NGTDM-DS	Neighbouring Grey Tone Difference Matrix dataset
WFS	Whole Feature Set
SMOTE	Synthetic Minority Over-sampling Technique
ADASYN	Adaptive Synthetic Sampling
ENN	Edited Nearest Neighbour
SMOTETOMEK	SMOTE and Tomek-Links
SMOTEENN	SMOTE and ENN
LR	Logistic Regression
SVM	Support Vector Machine
XGB	Extreme Gradient Boosting or XGBoost
RBF	Radial Basis Function
AUC ROC	Area under the Receiver Operating Characteristic Curve

## Appendix A

**Table A1:** Training results and hyperparameter optimisation for LR.

Dataset	Resampling Method	C	Regularizer	Best Solver	Mean F-Score	Std F-Score
GLRLM-DS	UNDERSAMPLING	1000	l1	liblinear	0.486	0.045
GLSZM-DS	UNDERSAMPLING	1000	l1	liblinear	0.402	0.109
GLRLM-DS	SMOTE	1000	l2	lbfgs	0.790	0.013

**Table A2:** Training results and hyperparameter optimisation for SVM.

Dataset	Resampling Method	Kernel	C	Gamma	Mean F-Score	Std F-Score
WFS	UNDERSAMPLING	linear	10	1	0.639	0.084
FOIS-DS	UNDERSAMPLING	linear	0.001	0.250	0.417	0.027
GLCM-DS	UNDERSAMPLING	poly	0.1	8	0.405	0.002
GLDM-DS	UNDERSAMPLING	poly	10	1	0.494	0.023
NGTDM-DS	UNDERSAMPLING	rbf	100	0.25	0.439	0.003
GLDM-GLRLM-DS	UNDERSAMPLING	linear	100	0.25	0.494	0.051
GLDM-GLSZM-GLRLM-DS	UNDERSAMPLING	linear	100	0.250	0.465	0.054
WFS	SMOTE	rbf	1000	auto	0.928	0.043
GLCM-DS	SMOTE	rbf	1000	0.25	0.837	0.012
GLSZM-DS	SMOTE	rbf	100	0.5	0.892	0.000
GLDM-DS	SMOTE	rbf	100	1	0.900	0.010
GLDM-GLRLM-DS	SMOTE	rbf	1000	auto	0.918	0.005
GLDM-GLSZM-GLRLM-DS	SMOTE	rbf	100	0.25	0.923	0.000
WFS	ADASYN	rbf	100	scale	0.886	0.012
GLCM-DS	ADASYN	rbf	100	0.25	0.845	0.000
GLRLM-DS	ADASYN	rbf	100	0.25	0.905	0.003
GLSZM-DS	ADASYN	rbf	100	0.5	0.891	0.004
GLDM-DS	ADASYN	rbf	10	1	0.893	0.012
GLDM-GLRLM-DS	ADASYN	rbf	10	0.5	0.924	0.001
GLDM-GLSZM-GLRLM-DS	ADASYN	rbf	10	0.5	0.922	0.004
WFS	SMOTETOMEK	rbf	100	scale	0.938	0.003
GLRLM-DS	SMOTETOMEK	rbf	10	4	0.917	0.006
GLSZM-DS	SMOTETOMEK	rbf	10	0.5	0.877	0.016
GLDM-DS	SMOTETOMEK	rbf	10	0.5	0.905	0.008
GLDM-GLRLM-DS	SMOTETOMEK	rbf	100	scale	0.921	0.003
GLDM-GLSZM-GLRLM-DS	SMOTETOMEK	rbf	10	1	0.928	0.015
WFS	SMOTEENN	rbf	1000	auto	0.914	0.003
GLRLM-DS	SMOTEENN	rbf	100	1	0.935	0.002
GLDM-DS	SMOTEENN	rbf	100	0.5	0.958	0.003
GLDM-GLRLM-DS	SMOTEENN	rbf	100	auto	0.9317	0.001
GLDM-GLSZM-GLRLM-DS	SMOTEENN	rbf	10	0.5	0.97	0.002

**Table A3:** Training results and hyperparameter optimisation for XGB.

Dataset	Resampling Method	n Estimators	Max Depth	lr	Mean F-Score	Std F-Score
FOIS-DS	SMOTE	500	4	0.1	0.893	0.014
FOIS-DS	ADASYN	500	4	0.1	0.892	0.008
NGTDM-DS	ADASYN	500	10	0.3	0.838	0.022
FOIS-DS	SMOTETOMEK	500	10	0.3	0.885	0.023
GLCM-DS	SMOTETOMEK	500	10	0.1	0.861	0.005
NGTDM-DS	SMOTETOMEK	500	10	0.1	0.840	0.021
FOIS-DS	SMOTEENN	500	6	0.3	0.894	0.027
GLCM-DS	SMOTEENN	500	10	0.1	0.924	0.008
GLSZM-DS	SMOTEENN	500	10	0.1	0.898	0.018
NGTDM-DS	SMOTEENN	500	10	0.1	0.918	0.012
